# Impact of Antipseudomonal Antibiotics in Patients with Bronchiectasis Who Experienced Exacerbation or Developed Pneumonia: A Nationwide Study in Japan

**DOI:** 10.3390/antibiotics13121182

**Published:** 2024-12-05

**Authors:** Akihiko Hagiwara, Hisayuki Shuto, Ryohei Kudoh, Shota Omori, Kazufumi Hiramatsu, Jun-ichi Kadota, Kiyohide Fushimi, Kosaku Komiya

**Affiliations:** 1 Respiratory Medicine and Infectious Diseases, Oita University Faculty of Medicine, 1-1 Idaigaoka, Hasama-machi, Yufu 879-5593, Oita, Japan; 2 Department of Health Policy and Informatics, Tokyo Medical and Dental University Graduate School, 1-5-45 Yushima, Bunkyo-ku, Tokyo 113-8519, Japan; 3 Research Center for GLOBAL and LOCAL Infectious Diseases, Oita University Faculty of Medicine, 1-1 Idaigaoka, Hasama-machi, Yufu 879-5593, Oita, Japan

**Keywords:** bronchiectasis, *Pseudomonas aeruginosa*, antibiotic, pneumonia, exacerbation of bronchiectasis

## Abstract

**Background/Objectives**: Although chronic infection by *Pseudomonas aeruginosa* among patients with bronchiectasis is associated with poor prognosis, the impact of antibiotics with *P. aeruginosa* coverage in patients with bronchiectasis who experienced bacterial pneumonia or exacerbation of bronchiectasis has not been fully investigated. **Methods**: This study targeted patients with bronchiectasis who were admitted to hospitals because of bacterial pneumonia or exacerbation of bronchiectasis between April 2018 and March 2020 using a national inpatient database in Japan. The association of antipseudomonal antibiotic treatment with in-hospital mortality was assessed after propensity score matching to adjust the patients’ backgrounds. **Results**: In total, 4943 patients with bacterial pneumonia and 1914 patients with exacerbation of bronchiectasis were included in this study. The in-hospital mortality rate did not differ between patients who did and did not receive antipseudomonal agents among patients with bacterial pneumonia (9.0% [185/2045] vs. 7.4% [151/2045]; *p* = 0.053) and those with exacerbation of bronchiectasis (5.2% [42/803] vs. 4.1% [33/803] group; *p* = 0.287). **Conclusions**: The use of antibiotics covering *P. aeruginosa* does not apparently improve prognosis in patients with bacterial pneumonia or exacerbation of bronchiectasis. A prospective study focusing on the impact of antibiotics covering *P. aeruginosa* among patients with bronchiectasis in whom *P. aeruginosa* is isolated is required.

## 1. Introduction

Bronchiectasis is a chronic disease defined radiologically by permanent and abnormal airway dilation and clinically by productive cough, sputum production, and recurrent infectious exacerbations [1]. The disease concept of bronchiectasis includes heterogeneous conditions with various underlying diseases. A previous systematic review of 8608 patients with bronchiectasis found that idiopathic bronchiectasis was the most common underlying condition (44.8%), followed by post-infection (29.9%), immunodeficiency (5.0%), chronic obstructive pulmonary disease (3.9%), and connective tissue disease (3.8%) [2]. However, there is some geographical variation in the incidence, prevalence, and clinical features of bronchiectasis [3,4,5]. Infection by nontuberculous mycobacteria is the most common background in patients with bronchiectasis in Japan and the United States [6,7]. The prevalence of bronchiectasis is increasing globally, and it is reportedly higher among women and older people. One study noted that the prevalence of bronchiectasis in the United Kingdom increased from 350.5 per 100,000 in 2004 to 566.1 per 100,000 in 2013 in women and from 301.2 per 100,000 in 2004 to 485.5 per 100,000 in 2013 in men [8]. Similar results have been reported from other countries [9,10,11].

The management objectives for patients with bronchiectasis are to reduce the frequency of exacerbations to prevent disease progression and improve prognosis. Airway clearance is a key treatment in bronchiectasis management. The British Thoracic Society (BTS) and European Respiratory Society (ERS) guidelines for the management of bronchiectasis in adults recommend long-term oral macrolides or inhaled antibiotics in patients with three or more exacerbations per year in addition to airway clearance [12,13]. Recently, studies focusing on the pathobiological relationships between bronchiectasis and *Pseudomonas aeruginosa* have been increasingly highlighted [14]. *P. aeruginosa* is frequently isolated from respiratory samples of patients with bronchiectasis. The European Multicentre Bronchiectasis Audit and Research Collaboration, an international research network for bronchiectasis in Europe, revealed that 3047 of 12,152 patients (25.1%) with bronchiectasis had positive cultures for *P. aeruginosa* [4]. Chronic *P. aeruginosa* infection has been linked to poor prognoses, including increases in exacerbations, hospital admissions, and mortality [15]. The BTS guidelines recommend regular sputum microbiology screening for patients with clinically significant bronchiectasis to detect pathogens, including newly isolated *P. aeruginosa*, suggesting treatment with antipseudomonal antibiotics for exacerbation of bronchiectasis if *P. aeruginosa* was detected in previous tests [13]. Furthermore, the ERS guidelines suggest that adults with bronchiectasis with a new isolation of *P. aeruginosa* should be offered eradication antibiotic treatment despite the extremely low quality of evidence [12].

Antibiotics covering *P. aeruginosa* are used for patients with bronchiectasis who experience bacterial pneumonia or exacerbation of bronchiectasis, especially when *P. aeruginosa* is isolated from respiratory samples. However, the impact of antibiotics with *P. aeruginosa* coverage in patients with bronchiectasis has not been fully elucidated. Thus, the effects of such antibiotics on prognosis among patients with bronchiectasis must be clarified to ensure proper antibiotic use. Therefore, we collected medical data on patients with bronchiectasis hospitalized because of bacterial pneumonia or exacerbation of bronchiectasis and evaluated the effectiveness of antipseudomonal therapy using the Japanese Diagnosis Procedure Combination (DPC) database to identify the association between the use of antipseudomonal antibiotics and mortality.

## 2. Results

### 2.1. Patients’ Characteristics

**Figure 1:**
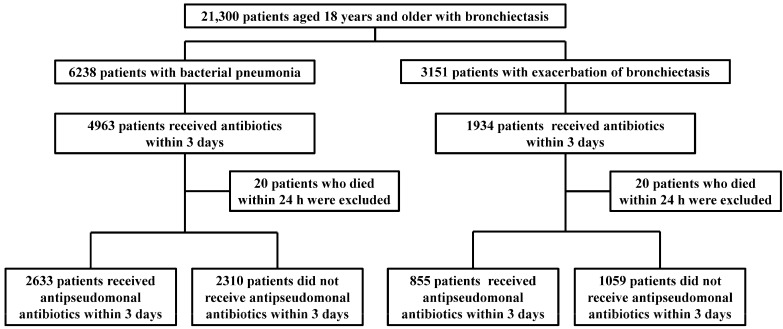
Flow chart of patient selection.

We identified 21,300 patients aged 18 years or older with bronchiectasis with an International Classification of Diseases, 10th Revision (ICD-10) code of A162 or J47 between April 2018 and March 2020. After excluding patients who did not receive any antibiotics within 3 days after admission or who died within 24 h after admission, 4943 patients admitted for bacterial pneumonia and 1914 patients admitted for exacerbation of bronchiectasis were included in this study (Figure 1). Of patients with bacterial pneumonia, 2633 and 2310 were classified into the antipseudomonal and nonantipseudomonal groups, respectively, and among those with exacerbation of bronchiectasis, 855 and 1059 were assigned to the antipseudomonal and nonantipseudomonal groups, respectively.

Among patients with bacterial pneumonia, the use of antipseudomonal antibiotics was linked to older age, female sex, lower body mass index (BMI), a higher Barthel index, a lower Charlson comorbidity index, and a higher Hugh–Jones dyspnea scale. Similarly, antipseudomonal antibiotics were more commonly used by patients without impaired consciousness; those who received supplemental oxygen, mechanical ventilation, or renal replacement therapy; those admitted to the intensive care unit (ICU); and those treated with antifungals, inhaled drugs, or vasopressors (Table 1). Meanwhile, among patients with exacerbation of bronchiectasis, antipseudomonal antibiotics were more frequently administered to those with lower BMI, a higher Charlson comorbidity index, and a higher Hugh–Jones dyspnea scale as well as those treated with antifungals or inhaled drugs (Table 2).

### 2.2. Propensity Score Matching (PSM)

A propensity score was calculated from all variables, and the area under the curve was 0.628 in patients with bacterial pneumonia and 0.609 in patients with exacerbation of bronchiectasis. After PSM, the baseline characteristics were well balanced between the groups for both patients with bacterial pneumonia and exacerbation of bronchiectasis.

### 2.3. Outcomes

In patients with bacterial pneumonia, the in-hospital mortality rate did not differ between the antipseudomonal and nonantipseudomonal groups (9.0% [185/2045] vs. 7.4% [151/2045]; *p* = 0.053), as presented in Table 3. Similarly, the length of hospital stay (15 days [interquartile range (IQR) = 10–24] vs. 14 days [IQR = 9–23]; *p* = 0.875) and total hospitalization costs (4023 US dollars [IQR = 2840–6273] vs. 3643 US dollars [IQR = 2598–5730]; *p* = 0.053) did not significantly differ between the groups. When patients were divided according to the timing of antibiotic treatment initiation, the in-hospital mortality rate among patients who received antibiotic treatment within 1 day after admission was higher in the antipseudomonal group than in the nonantipseudomonal group (9.1% [185/2041] vs. 7.1% [142/1989]; *p* = 0.025), whereas the mortality rate among patients who received antibiotic treatment 2 or 3 days after admission was higher in the nonantipseudomonal group than in the antipseudomonal group (16.1% [9/56] vs. 0% [0/4)].

In patients with exacerbation of bronchiectasis, the in-hospital mortality rate also did not differ between the antipseudomonal and nonantipseudomonal groups (5.2% [42/803] vs. 4.1% [33/803]; *p* = 0.287), as illustrated in Table 4. Although total hospitalization costs did not significantly differ between the groups (4029 US dollars [IQR = 2814–5909] vs. 3610 US dollars [IQR = 2202–5721]; *p* = 0.076), the length of hospital stay was significantly longer in the antipseudomonal group than in the nonantipseudomonal group (15 days [IQR = 10–22] vs. 12 days [IQR = 7–19]; *p* < 0.032). The in-hospital mortality rate also did not differ between patients categorized by the timing of antibiotic treatment initiation. Specifically, among patients who received antibiotic treatment within 1 day after admission, the in-hospital mortality rate was 4.8% (34/704) in the antipseudomonal group and 4.2% (26/622) in the nonantipseudomonal group (*p* = 0.570), whereas among patients who received antibiotic treatment 2 or 3 days after admission, the in-hospital mortality rates in the antipseudomonal and nonantipseudomonal groups were 8.1% (8/99) and 3.9% (7/181), respectively (*p* = 0.134).

Focusing on the patients’ ages and the types of antipseudomonal antibiotics, the in-hospital mortality rate increased with increasing age, and the in-hospital mortality rate was generally high among patients treated with carbapenems or tazobactam/piperacillin. Meanwhile, the in-hospital mortality rate was low among patients treated with ceftazidime or fluoroquinolones (Supplementary Tables S1 and S2).

## 3. Discussion

This study revealed that the use of antipseudomonal antibiotics was not associated with in-hospital mortality and total hospitalization costs among patients with bronchiectasis who developed bacterial pneumonia or experienced exacerbation of bronchiectasis. Although the length of hospital stay was significantly longer in the antipseudomonal group than in the nonantipseudomonal group among patients with exacerbation of bronchiectasis, no difference was noted among patients with bacterial pneumonia.

The use of antibiotics covering *P. aeruginosa* has been recommended for patients with critical conditions attributable to pneumonia, but recent studies failed to identify a benefit of antibiotics with *P. aeruginosa* coverage even for severe pneumonia [16,17,18]. Along with the severity of pneumonia, risk factors for antimicrobial resistance are considered for the indication of broad-spectrum antibiotics in clinical practice for pneumonia treatment. Although healthcare-associated pneumonia (HCAP) was first described as a concept of pneumonia in patients with frequent healthcare exposure who had a higher risk of infection by drug-resistant organisms, such as *P. aeruginosa* and methicillin-resistant *Staphylococcus aureus* (MRSA) [19], the use of antibiotics covering these organisms has not conferred favorable effects on clinical outcomes in patients with HCAP [16,17]. The American Thoracic Society and Infectious Diseases Society of America guidelines for community-acquired pneumonia (CAP) published in 2019 recommend against using the categorization of HCAP to guide the selection of extended-coverage antibiotics in patients with CAP and to select treatment that empirically covers *P. aeruginosa* or MRSA if they were isolated in previous tests [20]. However, a recent study demonstrated that the use of antibiotics covering *P. aeruginosa* in older adults who were admitted for CAP and in whom *P. aeruginosa* was isolated from sputum did not improve their prognosis [21]. The gap between the recommendations and real-world results could be linked to whether the isolation of *P. aeruginosa* from sputum reflects infection or colonization, as observed for pneumonia in patients in whom MRSA was isolated from respiratory samples [22].

*P. aeruginosa* is a major pathogen commonly isolated from the respiratory samples of patients with bronchiectasis [4], and it has been linked to poor prognoses [15,23]. Although the current study targeted patients with bronchiectasis, the use of antibiotics covering *P. aeruginosa* did not significantly decrease in-hospital mortality, suggesting that *P. aeruginosa* from respiratory samples might reflect colonization rather than infection. However, we could not identify the patients in whom *P. aeruginosa* was or was not isolated from respiratory samples because microbiological information was not contained in the DPC database. A study focusing on the impact of antibiotics covering *P. aeruginosa* only among patients with bronchiectasis in whom *P. aeruginosa* was isolated is required.

Similarly, the use of antipseudomonal antibiotics did not improve the prognosis of patients with exacerbation of bronchiectasis. However, whether attending physicians distinguished between bacterial pneumonia and exacerbation of bronchiectasis among patients in the DPC database remains unclear. As observed for bacterial pneumonia [21], the isolation of *P. aeruginosa* from respiratory samples might not always reflect respiratory infection. The international guidelines for bronchiectasis management recommend long-term oral macrolides or inhaled antibiotics in patients with three or more exacerbations per year [12,13]. Additionally, eradication antibiotic treatment is recommended for patients with bronchiectasis in whom *P. aeruginosa* is newly isolated despite an extremely low quality of evidence [12], and a recent study revealed that eradication treatment with combined systemic and inhaled antibiotics achieved higher eradication rates than systemic antibiotic alone [24]. For acute exacerbation of bronchiectasis, the BTS guidelines recommend the use of antibiotics covering *P. aeruginosa* if *P. aeruginosa* was detected in previous tests, and the ERS guidelines recommend antibiotic therapy for 14 days [12,13]. The current results suggest that patients with exacerbation of bronchiectasis do not necessarily require treatment with antibiotics covering *P. aeruginosa*. The need for antibiotics covering *P. aeruginosa* should be carefully determined on the basis of patients’ backgrounds and the organisms detected in previous respiratory sample cultures in consideration of whether the isolated pathogens reflect colonization or infection [21,25,26].

In the subgroup analyses, the in-hospital mortality rate among patients who received antibiotic treatment within 1 day after admission was higher in the antipseudomonal group than in the nonantipseudomonal group, whereas the mortality rate among patients who received antibiotic treatment 2 or 3 days after admission was likely higher in the nonantipseudomonal group than in the antipseudomonal group. Meanwhile, the mortality rate was higher in patients treated with carbapenems or tazobactam/piperacillin than in those treated with other antipseudomonal antibiotics. However, the timing of treatment initiation and type of antipseudomonal antibiotics were not included as adjustment factors in PSM. Therefore, we could not identify the optimal treatment timing or antipseudomonal antibiotic type for patients who developed bacterial pneumonia or experienced exacerbation of bronchiectasis. Factors not provided by the DPC database might have confounded these associations.

The strength of this study is that we focused on the association between the use of antipseudomonal antibiotics and in-hospital mortality among patients with bronchiectasis who were admitted for bacterial pneumonia or exacerbation of bronchiectasis using a national inpatient database in Japan. We adjusted the patients’ backgrounds by PSM because patients with severe pneumonia might be treated with antibiotics covering *P. aeruginosa*.

However, the current study had several limitations. First, the reliability and validity of the diagnosis recorded with an ICD-10 code are not strictly guaranteed even when the DPC database is used. The diagnoses of bacterial pneumonia and exacerbation of bronchiectasis in the current study were based on the “admission-precipitating diagnosis” in the DPC database, which was determined by attending physicians. Physicians might not accurately distinguish the exacerbation of bronchiectasis from bacterial pneumonia. Whether the current patients met the international diagnostic criteria also remains uncertain because we could not obtain data on clinical symptoms and radiological features [27]. Moreover, the causative underlying diseases for the development of bronchiectasis were not identified. Second, it is unclear whether *P. aeruginosa* was detected in respiratory samples because the DPC database does not contain microbiological data. Because of the same reasons, the information regarding the drug susceptibility patterns of *P. aeruginosa* could not be obtained. Additionally, we could not evaluate the risk factors for antimicrobial resistance among individual patients [28,29]. Third, only limited outcomes could be assessed using the DPC database. Although we examined in-hospital mortality, length of hospital stay, and total hospitalization costs for outcomes, other outcomes and variables, such as clinical cure, the amount of supplemental oxygen, and disease severity, could not be evaluated. Fourth, this study focused on whether antibiotics covering *P. aeruginosa* were administered within 3 days after admission; thus, the effects of escalation or de-escalation therapy on prognosis was not investigated. Finally, it remains uncertain whether each patient received an adequate dosage of antibiotics. The DPC database lacks serological data, including renal and liver functions, to calculate the appropriate dosage of antibiotics.

## 4. Methods

### 4.1. Data Source

This retrospective cohort study used the DPC database, a national inpatient database in Japan. The DPC system has been adopted by 1764 hospitals (approximately 480,000 beds) as of 2022, and it covers most hospital admissions for acute illnesses in Japan [30,31]. This database contains patient data on sex, age, body height and weight, smoking history (Brinkman index), activities of daily living score (Barthel index), state of consciousness (Japan Coma Scale) on admission, Charlson comorbidity index, Hugh–Jones dyspnea scale, diagnoses (“main diagnosis”, “admission-precipitating diagnosis”, “most resource-consuming diagnosis”, “second most resource-consuming diagnosis”, “comorbidities present at time of admission”, and “conditions arising after admission”) recoded with ICD-10 codes, procedures, drug administration, ICU admission, length of hospitalization, total hospitalization costs, and discharge status.

This study was approved by the Institutional Ethics Committee of Oita University Faculty of Medicine (approval no. 2765, 22 March 2024). The requirement for informed consent was waived by the committee because of the retrospective design of the study. Information for this research was posted at the hospital.

### 4.2. Study Population

Patients with bronchiectasis who were admitted to DPC hospitals because of bacterial pneumonia or exacerbation of bronchiectasis between April 2018 and March 2020 were included in this study. We first extracted patients with an ICD-10 code of A162 or J47, which indicates bronchiectasis as their main diagnosis, admission-precipitating diagnosis, most resource-consuming diagnosis, or second-most resource-consuming diagnosis or as a comorbidity present during admission. Next, patients with bronchiectasis hospitalized because of bacterial pneumonia or exacerbation of bronchiectasis were identified using the “admission-precipitating diagnosis” recorded using an ICD-10 code and Japanese disease names. Patients were grouped according to the receipt of antipseudomonal antibiotics (antipseudomonal antibiotic group or nonantipseudomonal antibiotic group), to which *P. aeruginosa* is generally considered susceptible, within 3 days after admission. Patients who did not receive any antibiotics within 3 days of admission, those younger than 18 years, and those who died within 24 h after admission were excluded. Our prior study focused on the medical causes of hospitalization among all patients with bronchiectasis using the DPC data of hospitalized patients with bronchiectasis between April 2018 and March 2020 [32]. However, the current study only targeted patients who were admitted for pneumonia or exacerbation of bronchiectasis, as this study’s aim was fundamentally different from that of the previous epidemiology study.

### 4.3. Data Collection and Outcomes

Patients’ background information, including sex, age, BMI, Brinkman index, Barthel index, Japan Coma Scale on admission, Charlson comorbidity index, Hugh–Jones dyspnea scale, length of hospital stay, and total hospitalization costs (1 US dollar equivalent to 150 Japanese yen), was collected from the DPC database. BMI was categorized using the following ranges in accordance with the World Health Organization definitions: <18.5, 18.5–24.9, 25.0–29.9, and ≥30.0 kg/m^2^ [33]. The Barthel index was classified into three categories: 0–55, 60–95, and 100. The Charlson comorbidity index was classified into four categories: 0, 1–2, 3–4, and ≥5. The Japan Coma Scale is widely used to assess patients’ consciousness level in Japan, and it is considerably correlated with the Glasgow Coma Scale [34].

To clarify the receipt of antipseudomonal antibiotics within 3 days of admission, the use of antibiotics listed in The Japanese Respiratory Society Guidelines for the Management of Pneumonia in Adults 2024 was investigated [35]. Specifically, piperacillin, tazobactam/piperacillin, ceftazidime, cefepime, cefozopran, cefpirome, imipenem/cilastatin, panipenem/betamipron, meropenem, biapenem, doripenem, tosufloxacin, levofloxacin, sitafloxacin, ciprofloxacin, pazufloxacin mesylate, gentamicin, amikacin, and tobramycin were considered antipseudomonal antibiotics [35,36].

In addition to antibiotics, the following drug administrations and procedures within 7 days were also surveyed: antifungals, including amphotericin B, liposomal amphotericin B, micafungin, caspofungin acetate, fluconazole, fosfluconazole, itraconazole, and voriconazole; inhaled drugs, including inhaled corticosteroids, long-acting β2 agonists, long-acting muscarinic antagonists, combinations of inhaled corticosteroids and long-acting β2 agonists, combinations of long-acting β2 agonists, long-acting muscarinic antagonists, and inhaled corticosteroids, combinations of long-acting β2 agonists and long-acting muscarinic antagonists, short-acting β2 agonists, and short-acting muscarinic antagonists; intravenous vasopressors, including dopamine, dobutamine, noradrenaline, and adrenaline; transfusions, including red blood cell transfusion, fresh frozen plasma transfusion, and platelet transfusion; supplemental oxygen; mechanical ventilation; renal replacement therapy; extracorporeal membrane oxygenation; and ICU admission.

The primary endpoint was in-hospital mortality, and the secondary endpoints were the length of hospitalization and total hospitalization costs.

### 4.4. PSM

To adjust the baseline characteristics of patients, we used 1:1 PSM with a nearest-neighbor method and set the caliper as 0.2. The propensity score was calculated using a logistic regression model with the following covariates: age, sex, BMI, smoking history, Barthel index, Japan Coma Scale on admission, Charlson comorbidity index, Hugh–Jones dyspnea scale, and treatment within 7 days after admission (antifungals, inhaled drugs, intravenous vasopressors, transfusion, supplemental oxygen, mechanical ventilation, renal replacement therapy, extracorporeal membrane oxygenation, and ICU admission).

### 4.5. Statistical Analysis

Statistical analyses were performed using the Statistical Package for the Social Sciences version 22 (IBM, Armonk, NY, USA). We performed comparisons between the two groups using a *t*-test for continuous variables and a chi-squared test or Fisher’s exact test for categorical variables. *p*-values smaller than 0.05 were considered statistically significant. Additionally, subgroup analyses focusing on the timing of treatment (within 1 day of admission vs. 2 or 3 days after admission), age, and the types of antipseudomonal antibiotics were conducted.

## 5. Conclusions

Although bronchiectasis is known to be associated with the isolation of *P. aeruginosa* from respiratory samples, the use of antibiotics covering *P. aeruginosa* for bacterial pneumonia and exacerbation of bronchiectasis does not necessarily contribute to an improved prognosis. A prospective study focusing on the impact of antibiotics covering *P. aeruginosa* among patients with bronchiectasis in whom *P. aeruginosa* was isolated is required.

## Figures and Tables

**Table 1:** Clinical characteristics of patients with bronchiectasis before and after propensity score matching (admitted for bacterial pneumonia).

	Unmatched			Matched		
	Antipseudomonal Group (n = 2633)	Nonantipseudomonal Group (n = 2310)	*p*	Antipseudomonal Group (n = 2045)	Nonantipseudomonal Group (n = 2045)	*p*
Age (years)	78 (71–84)	81 (73–86)	<0.001	79 (72–85)	80 (72–86)	0.557
Sex (female)	1796 (68.2)	1507 (65.2)	0.027	1369 (66.9)	1355 (66.3)	0.643
Body mass index (kg/m^2^)						
<18.5	1388 (52.7)	1077 (46.6)	<0.001	1004 (49.1)	1004 (49.1)	1.000
18.5–24.9	951 (36.1)	899 (38.9)	0.042	780 (38.1)	784 (38.3)	0.898
25.0–29.9	114 (4.3)	105 (4.5)	0.713	96 (4.7)	96 (4.7)	1.000
≥30.0	16 (0.6)	28 (1.2)	0.024	15 (0.7)	14 (0.7)	0.852
Missing data	164 (6.2)	201 (8.7)	0.001	150 (7.3)	147 (7.2)	0.857
Smoking history						
Nonsmoker	1906 (72.4)	1687 (73.0)	0.614	1493 (73.0)	1489 (72.8)	0.888
Current/past smoker	469 (17.8)	428 (18.5)	0.515	366 (17.9)	377 (18.4)	0.656
Missing data	258 (9.8)	195 (8.4)	0.099	186 (9.1)	179 (8.8)	0.701
Barthel index						
0–55	792 (30.1)	771 (33.4)	0.013	641 (31.3)	638 (31.2)	0.919
60–95	447 (17.0)	390 (16.9)	0.930	351 (17.2)	346 (16.9)	0.835
100	1026 (39.0)	846 (36.6)	0.090	778 (38.0)	787 (38.5)	0.772
Missing data	368 (14.0)	303 (13.1)	0.379	275 (13.4)	274 (13.4)	0.963
Charlson comorbidity index						
0	12 (0.5)	17 (0.7)	0.198	11 (0.5)	10 (0.5)	0.827
1–2	1931 (73.3)	1522 (65.9)	<0.001	1433 (70.1)	1421 (69.5)	0.683
3–4	613 (23.3)	689 (29.8)	<0.001	533 (26.1)	542 (26.5)	0.749
≥5	77 (2.9)	82 (3.5)	0.214	68 (3.3)	72 (3.5)	0.731
Japan Coma Scale						
0	2282 (86.7)	1925 (83.3)	0.001	1741 (85.1)	1748 (85.5)	0.757
1–3	289 (11.0)	330 (14.3)	<0.001	259 (12.7)	257 (12.6)	0.925
10–30	45 (1.7)	48 (2.1)	0.341	39 (1.9)	35 (1.7)	0.639
100–300	17 (0.6)	7 (0.3)	0.084	6 (0.3)	5 (0.2)	0.763
Hugh–Jones dyspnea scale						
1	376 (14.3)	365 (15.8)	0.135	321 (15.7)	322 (15.7)	0.966
2	428 (16.3)	357 (15.5)	0.442	336 (16.4)	334 (16.3)	0.933
3	393 (14.9)	304 (13.2)	0.075	276 (13.5)	286 (14.0)	0.650
4	513 (19.5)	422 (18.3)	0.276	386 (18.9)	384 (18.8)	0.936
5	442 (16.8)	332 (14.4)	0.020	302 (14.8)	304 (14.9)	0.930
Missing data	481 (18.3)	530 (22.9)	<0.001	424 (20.7)	415 (20.3)	0.727
Supplemental oxygen	1572 (59.7)	1168 (50.6)	<0.001	1089 (53.3)	1088 (53.2)	0.975
Mechanical ventilation	178 (6.8)	57 (2.5)	<0.001	57 (2.8)	57 (2.8)	1.000
Antifungal	97 (3.7)	25 (1.1)	<0.001	27 (1.3)	25 (1.2)	0.780
Inhaled drug	633 (24.0)	431 (18.7)	<0.001	417 (20.4)	412 (20.1)	0.846
Vasopressor	72 (2.7)	26 (1.1)	<0.001	28 (1.4)	25 (1.2)	0.678
Red blood cell transfusion	33 (1.3)	20 (0.9)	0.187	17 (0.8)	20 (1.0)	0.620
Fresh frozen plasma transfusion	2 (0.1)	2 (0.1)	1	0 (0.0)	1 (0.0)	1.000
Platelet transfusion	3 (0.1)	1 (0.0)	0.628	0 (0.0)	1 (0.0)	1.000
Renal replacement therapy	24 (0.9)	7 (0.3)	0.007	11 (0.5)	7 (0.3)	0.345
ICU admission	33 (1.3)	10 (0.4)	0.002	7 (0.3)	10 (0.5)	0.466

Values are presented as the median (interquartile range) or n (%). ICU, intensive care unit.

**Table 2:** Clinical characteristics of patients with bronchiectasis before and after propensity score matching (admitted for exacerbation of bronchiectasis).

	Unmatched			Matched		
	Antipseudomonal Group (n = 855)	Nonantipseudomonal Group (n = 1059)	*p*	Antipseudomonal Group (n = 803)	Nonantipseudomonal Group (n = 803)	*p*
Age (years)	75 (68–81)	76 (68–82)	0.645	75 (68–82)	76 (68–82)	0.802
Sex (female)	618 (72.3)	770 (72.7)	0.834	577 (71.9)	593 (73.8)	0.369
Body mass index (kg/m^2^)						
<18.5	449 (52.5)	506 (47.8)	0.039	425 (52.9)	422 (52.6)	0.881
18.5–24.9	312 (36.5)	431 (40.7)	0.060	288 (35.9)	288 (35.9)	1.000
25.0–29.9	41 (4.8)	55 (5.2)	0.691	39 (4.9)	44 (5.5)	0.573
≥30.0	11 (1.3)	10 (0.9)	0.475	11 (1.4)	10 (1.2)	0.826
Missing data	42 (4.9)	57 (5.4)	0.644	40 (5.0)	39 (4.9)	0.908
Smoking history						
Nonsmoker	653 (76.4)	789 (74.5)	0.345	610 (76.0)	624 (77.7)	0.408
Current/past smoker	145 (17.0)	173 (16.3)	0.716	136 (16.9)	128 (15.9)	0.590
Missing data	57 (6.7)	97 (9.2)	0.046	57 (7.1)	51 (6.4)	0.550
Barthel index						
0–55	170 (19.9)	226 (21.3)	0.434	163 (20.3)	163 (20.3)	1.000
60–95	151 (17.7)	181 (17.1)	0.744	146 (18.2)	148 (18.4)	0.897
100	420 (49.1)	528 (49.9)	0.749	390 (48.6)	389 (48.4)	0.960
Missing data	114 (13.3)	124 (11.7)	0.284	104 (13.0)	103 (12.8)	0.941
Charlson comorbidity index						
0	299 (35.0)	437 (41.3)	0.005	286 (35.6)	298 (37.1)	0.534
1–2	431 (50.4)	476 (44.9)	0.017	402 (50.1)	394 (49.1)	0.690
3–4	113 (13.2)	127 (12.0)	0.421	103 (12.8)	102 (12.7)	0.940
≥5	12 (1.4)	19 (1.8)	0.501	12 (1.5)	9 (1.1)	0.510
Japan Coma Scale						
0	785 (91.8)	978 (92.4)	0.664	738 (91.9)	739 (92.0)	0.927
1–3	61 (7.1)	67 (6.3)	0.482	58 (7.2)	56 (7.0)	0.846
10–30	8 (0.9)	10 (0.9)	0.985	7 (0.9)	8 (1.0)	0.795
100–300	1 (0.1)	4 (0.4)	0.388	0 (0.0)	0 (0.0)	
Hugh–Jones dyspnea scale						
1	171 (20.0)	285 (26.9)	<0.001	169 (21.0)	162 (20.2)	0.666
2	172 (20.1)	157 (14.8)	0.002	145 (18.1)	149 (18.6)	0.796
3	135 (15.8)	157 (14.8)	0.560	127 (15.8)	131 (16.3)	0.786
4	165 (19.3)	166 (15.7)	0.037	159 (19.8)	156 (19.4)	0.850
5	118 (13.8)	132 (12.5)	0.388	109 (13.6)	119 (14.8)	0.475
Missing data	94 (11.0)	162 (15.3)	0.006	94 (11.7)	86 (10.7)	0.527
Supplemental oxygen	467 (54.6)	555 (52.4)	0.335	437 (54.4)	432 (53.8)	0.802
Mechanical ventilation	67 (7.8)	61 (5.8)	0.071	60 (7.5)	52 (6.5)	0.433
Antifungal	30 (3.5)	21 (2.0)	0.039	26 (3.2)	18 (2.2)	0.221
Inhaled drug	235 (27.5)	225 (21.2)	0.001	202 (25.2)	202 (25.2)	1.000
Vasopressor	27 (3.2)	52 (4.9)	0.055	23 (2.9)	21 (2.6)	0.760
Red blood cell transfusion	14 (1.6)	19 (1.8)	0.793	13 (1.6)	13 (1.6)	1.000
Fresh frozen plasma transfusion	3 (0.4)	5 (0.5)	0.738	2 (0.2)	3 (0.4)	1.000
Platelet transfusion	2 (0.2)	1 (0.1)	0.589	1 (0.1)	1 (0.1)	1.000
Renal replacement therapy	9 (1.1)	13 (1.2)	0.721	9 (1.1)	9 (1.1)	1.000
ICU admission	17 (2.0)	19 (1.8)	0.756	14 (1.7)	13 (1.6)	0.846

Values are presented as the median (interquartile range) or n (%). ICU, intensive care unit.

**Table 3:** Outcomes before and after propensity score matching (admitted for bacterial pneumonia).

	Unmatched			Matched		
	Antipseudomonal Group (n = 2633)	Nonantipseudomonal Group (n = 2310)	*p*	Antipseudomonal Group (n = 2045)	Nonantipseudomonal Group (n = 2045)	*p*
In-hospital mortality	274 (10.4)	174 (7.5)	<0.001	185 (9.0)	151 (7.4)	0.053
Hospital stay (days)	15 (10–25)	14 (9–23)	0.308	15 (10–24)	14 (9–23)	0.875
Hospitalization cost (US dollars)	4165 (2919–6502)	3661 (2626–5712)	<0.001	4023 (2840–6273)	3643 (2598–5730)	0.053

Values are presented as the median (interquartile range) or n (%).

**Table 4:** Outcomes before and after propensity score matching (admitted for exacerbation of bronchiectasis).

	Unmatched			Matched		
	Antipseudomonal Group (n = 855)	Nonantipseudomonal Group (n = 1059)	*p*	Antipseudomonal Group (n = 803)	Nonantipseudomonal Group (n = 803)	*p*
In-hospital mortality	47 (5.5)	47 (4.4)	0.287	42 (5.2)	33 (4.1)	0.287
Hospital stay (days)	15 (10–22)	11 (7–18)	0.004	15 (10–22)	12 (7–19)	0.032
Hospitalization cost (US dollars)	4002 (2811–5838)	3457 (2157–5830)	0.039	4029 (2814–5909)	3610 (2202–5721)	0.076

Values are presented as the median (interquartile range) or n (%).

## Data Availability

The data are available from the corresponding author upon reasonable request.

## Supplementary Materials

The following supporting information can be downloaded at https://www.mdpi.com/article/10.3390/antibiotics13121182/s1: Table S1: In-hospital mortality rate in the antipseudomonal group according to age and the type of antipseudomonal antibiotics among patients admitted for bacterial pneumonia; Table S2: In-hospital mortality rate in the antipseudomonal group according to age and the type of antipseudomonal antibiotics among patients admitted for exacerbation of bronchiectasis.
