# Supplementary material for: Impact of Antipseudomonal Antibiotics in Patients with Bronchiectasis Who Experienced Exacerbation or Developed Pneumonia: A Nationwide Study in Japan
Source: Antibiotics (Basel). 2024 Dec 5;13(12):1182. doi: 10.3390/antibiotics13121182 (PMC11672765; doi:10.3390/antibiotics13121182)
Supplement: Supplementary file 1 [file antibiotics-13-01182-s001.zip › antibiotics-3294747-supplementary.pdf]

Supplementary Table S1. In-hospital mortality rate in the antipseudomonal group according to age and the type of antipseudomonal antibiotics among patients admitted for bacterial pneumonia

Age	Types of antipseudomonal antibiotics						
	All antipseudomonal antibiotics	Carbapenems	Tazobactam/piperacillin	Cefepime	Ceftazidime	Fluoroquinolones	Others
All patients	9.0 (185/2045)	16.1 (63/391)	9.8 (126/1281)	12.3 (20/163)	8.6 (20/233)	6.1 (27/443)	8.0 (8/100)
10s or 20s	0.0 (0/2)	none	0.0 (0/1)	none	none	none	0.0 (0/1)
30s	0.0 (0/10)	0.0 (0/3)	0.0 (0/7)	none	none	0.0 (0/3)	none
40s	7.1 (1/14)	50.0 (1/2)	0.0 (0/9)	0.0 (0/1)	0.0 (0/2)	0.0 (0/5)	none
50s	4.3 (2/46)	7.1 (1/14)	0.0 (0/25)	25.0 (1/4)	0.0 (0/8)	11.1 (1/9)	0.0 (0/2)
60s	4.2 (12/289)	8.8 (5/57)	5.4 (8/149)	10.0 (3/30)	0.0 (0/40)	5.9 (5/85)	0.0 (0/12)
70s	6.4 (45/705)	7.9 (10/127)	6.9 (30/432)	10.0 (7/70)	8.8 (8/91)	3.9 (6/153)	2.9 (1/34)
80s	11.8 (94/794)	24.0 (36/150)	12.9 (70/544)	13.0 (6/46)	10.7 (8/75)	5.8 (9/154)	11.6 (5/43)
over 90s	16.8 (31/185)	26.3 (10/38)	15.8 (18/114)	25.0 (3/12)	23.5 (4/17)	17.6 (6/34)	25.0 (2/8)

Values are presented as n (%).

Supplementary Table S2. In-hospital mortality rate in the antipseudomonal group according to age and the type of antipseudomonal antibiotics among patients admitted for exacerbation of bronchiectasis

Age	Types of antipseudomonal antibiotics						
	All antipseudomonal antibiotics	Carbapenems	Tazobactam/piperacillin	Cefepime	Ceftazidime	Fluoroquinolones	Others
All patients	5.2 (42/803)	7.2 (11/153)	8.2 (28/342)	5.9 (6/101)	0.8 (1/125)	4.5 (9/201)	3.9 (4/103)
10s or 20s	0.0 (0/5)	none	0.0 (0/2)	0.0 (0/2)	0.0 (0/1)	0.0 (0/2)	none
30s	0.0 (0/21)	0.0 (0/2)	0.0 (0/5)	0.0 (0/1)	0.0 (0/12)	0.0 (0/2)	0.0 (0/2)
40s	0.0 (0/15)	0.0 (0/2)	0.0 (0/2)	0.0 (0/1)	0.0 (0/4)	0.0 (0/3)	0.0 (0/6)
50s	3.6 (1/28)	0.0 (0/5)	9.1 (1/11)	0.0 (0/4)	11.1 (1/9)	0.0 (0/10)	0.0 (0/2)
60s	5.6 (9/162)	8.9 (4/45)	8.3 (5/60)	3.6 (1/28)	0.0 (0/20)	4.5 (2/44)	10.0 (2/20)
70s	4.6 (15/326)	3.4 (2/59)	6.8 (9/132)	7.0 (3/43)	0.0 (0/61)	7.3 (6/82)	2.4 (1/41)
80s	6.7 (14/209)	10.8 (4/37)	9.5 (11/116)	10.5 (2/19)	0.0 (0/12)	2.1 (1/47)	3.7 (1/27)
over 90s	8.1 (3/37)	33.3 (1/3)	14.3 (2/14)	0.0 (0/3)	0.0 (0/6)	0.0 (0/11)	0.0 (0/59)

Values are presented as n (%).
